# SD‐OCT‐based biomarkers in predicting treatment outcomes of macular oedema secondary to retinal vein occlusion treated with anti‐VEGF therapy

**DOI:** 10.1111/aos.17574

**Published:** 2025-08-04

**Authors:** Ken K. Tsang, Vivian W. K. Hui, Christopher M. K. Pang, Ziqi Tang, Dawei Yang, Truong X. Nguyen, Shaheeda Mohamed, Timothy Y. Y. Lai, Carol Y. Cheung, Simon K. H. Szeto

**Affiliations:** ^1^ Department of Ophthalmology and Visual Sciences The Chinese University of Hong Kong Hong Kong Special Administrative Region China; ^2^ Hong Kong Eye Hospital Hong Kong Special Administrative Region China; ^3^ 2010 Eye & Cataract Centre Hong Kong Special Administrative Region China; ^4^ Pao So Kok Macular Disease Treatment and Research Centre, The Chinese University of Hong Kong Hong Kong Special Administrative Region China

## Abstract

**Purpose:**

To investigate the role of spectral domain optical coherence tomography (SD‐OCT)‐based biomarkers in predicting treatment response of macular oedema (MO) secondary to retinal vein occlusion (RVO) to anti‐vascular endothelial growth factor (VEGF) therapy.

**Methods:**

Retrospective cohort study including consecutive cases of RVO associated MO who received anti‐VEGF injections between January 2020 and April 2021. LogMAR visual acuity (VA) at baseline, 12 and 24 months was correlated with a panel of SD‐OCT‐based biomarkers, including vitreomacular status, size of intra‐retinal cysts (IRC), presence of disorganization of retinal inner layers (DRIL), hyper‐reflective foci (HRF) in the retina, integrity of the external limiting membrane (ELM), ellipsoid zone (EZ), cone outer segment tip (COST) and presence of subretinal fluid (SRF).

**Results:**

One hundred and thirty eyes were included with 81 and 49 eyes in the BRVO and CRVO subgroup, respectively. In both subgroups, baseline disrupted EZ/ELM [BRVO: (*β* = 0.144 *p* = 0.008; *β* = 0.111 *p* = 0.014; *β* = 0.096 *p* = 0.042) and CRVO: (*β* = 0.316 *p* < 0.001; *β* = 0.336 *p* < 0.001; *β* = 0.327 *p* < 0.001)] were associated with worse VA from baseline through 24 months. In the BRVO subgroup, the presence of HRF (*β* = 0.209 *p* < 0.001) correlated with worse baseline VA. Improvement in DRIL extent [OR = 4.355 (1.109–17.094) *p* = 0.035; OR = 4.510 (1.707–11.917) *p* = 0.002] and EZ/ELM integrity [OR = 4.474 (1.783–11.223) *p* = 0.001; OR = 3.214 (1.414–7.305) *p* = 0.005] were associated with a higher likelihood of achieving at least a 5 letters gain at 12 and 24 months.

**Conclusion:**

A comprehensive system of SD‐OCT‐based features could predict functional outcomes of MO secondary to RVO with anti‐VEGF therapy up to 24 months.

## 1 INTRODUCTION

Retinal vein occlusion (RVO) is the second commonest retinal vascular disorder after diabetic retinopathy (DR), with macular oedema (MO) being the leading cause of vision loss (Laouri et al., 2011; Rogers, McIntosh, Cheung, et al., 2010; Rogers, McIntosh, Lim, et al., 2010). In RVO, capillary damage and retinal ischaemia led to elevated vitreous levels of vascular endothelial growth factors (VEGF), which promote vascular hyper‐permeability and leakage, resulting in the formation of MO (Tomkins‐Netzer et al., 2015).

Anti‐VEGF therapy is the standard‐of‐care treatment in RVO associated MO (RVO‐MO) (Rogers, McIntosh, Cheung, et al., 2010; Rogers, McIntosh, Lim, et al., 2010; Scott et al., 2017). Despite treatment, a substantial proportion of patients (40%–53%) gained less than 15 letters in best corrected visual acuity (BCVA) in randomized controlled trials (RCT) (Brown et al., 2010; Campochiaro et al., 2010, 2015; Holz et al., 2013). In the real‐world clinical settings, the mean gain in VA is less than that of RCT, which could be partially explained by suboptimal anti‐VEGF injection intensities and more heterogeneous patients with diverse clinical characteristics (Hattenbach et al., 2023). In view of the high treatment burden, there is a need for better prognostication, to facilitate effective patient counselling and resource allocation (Laouri et al., 2011).

Spectral‐domain optical coherence tomography (SD‐OCT) is a widely available and non‐invasive imaging tool that serves as the gold standard to diagnose and monitor macular diseases. Quantitative and qualitative OCT features are potential biomarkers to predict treatment response to anti‐VEGF in macular diseases, such as diabetic macular oedema (DMO) and RVO‐MO (Hui et al., 2022; Mao et al., 2022; Szeto et al., 2021, 2024). However, there is limited evidence on the predictive value of OCT biomarkers in RVO (Szeto et al., 2021; Szeto, Hui, Siu, et al., 2023; Szeto, Hui, Tang, et al., 2023). The presence of disorganization of retinal inner layers (DRIL) correlated with worse VA in eyes that underwent anti‐VEGF therapy (Babiuch et al., 2019; Mimouni et al., 2017). Other OCT parameters, such as epi‐retinal membrane (ERM), intraretinal cyst (IRC) size, thicker subretinal fluid (SRF), disrupted external limiting membrane (ELM), ellipsoid zone (EZ) and presence of hyper‐reflective retinal foci (HRF) are potential biomarkers that correlate with baseline VA (Castro‐Navarro et al., 2022; Yiu et al., 2020). Conversely, in the post‐hoc analysis of a RCT, Yiu et al. reported that none of these OCT‐based biomarkers were independently predictive of VA after 7 monthly ranibizumab injections (Yiu et al., 2020). The inconsistency in the literature could be explained by heterogeneity in study designs and statistical analysis. Hence, the role of OCT‐based biomarkers in predicting long‐term treatment response of RVO‐MO remains a critical knowledge gap and requires further investigation. Moreover, quantitative measurement of these biomarkers could be labour‐intensive and sometimes requires specialized software, limiting their application in real‐world retina clinics (Mimouni et al., 2017). Therefore, a comprehensive OCT‐based system that includes relevant biomarkers that are applicable in clinical settings is needed (Panozzo et al., 2020; Szeto et al., 2024).

In this study, we propose a comprehensive SD‐OCT‐based biomarkers system that is practical to use in a clinical setting (each biomarker is graded semi‐quantitatively into 2–3 levels) and investigate whether it could predict the long‐term anatomical and functional treatment response of RVO‐MO to anti‐VEGF therapy.

## 2 METHODS

This is a retrospective study performed in Hong Kong Eye Hospital, a tertiary ophthalmic centre in Hong Kong SAR, China. This study adhered to the Declaration of Helsinki and ethical approval was obtained from the local ethical committee.

Consecutive patients with RVO‐MO who received intravitreal anti‐VEGF injections of either aflibercept (Eylea, Bayer, Leverkusen, Germany) or ranibizumab (Lucentis, Novartis, Basel, Switzerland) between 1st January 2020 and 30th April 2021 were included. Treatment‐naïve patients would receive 3 loading doses of either aflibercept or ranibizumab, followed by pro re nata (PRN) injection based on VA and central subfield thickness (CST) (Tadayoni et al., 2016). Previously treated patients with recurrent MO would receive additional injections based on the same criteria. Standard fluorescein angiography (FA) (Spectralis, Heidelberg, Germany) or ultra‐wide field (UWF) FA (Mirante, Nidek, Japan) was performed in patients with clinical features of ischaemic RVO, such as the presence of a pupillary defect, diffuse cotton wool spots or retinal haemorrhages (Hayreh et al., 1990). The choice of FA device was based on the availability of the device. Laser pan‐retinal photocoagulation (PRP) would be performed in patients with signs of retinal ischaemia. Eyes which underwent previous vitreo‐retinal surgery were excluded from the investigation.

### 2.1 Study participants

Inclusion criteria were: (1) patients who were 18 years or older with either central RVO (CRVO), hemi‐RVO (HRVO) or branch RVO (BRVO); (2) presence of macular oedema, defined as presence of macular thickening and elevated CST (≥320 μm in male and ≥305 μm in female) with or without intra‐retinal or subretinal fluid (Sun et al., 2021); (3) received intravitreal anti‐VEGF injections during the study period.

Exclusion criteria were: (1) macular pathologies other than RVO‐associated MO, such as age‐related macular degeneration (AMD) and macular scar; (2) presence of retinopathy other than RVO, such as retinitis pigmentosa; (3) presence of pre‐existing vision‐threatening diabetic retinopathy (VTDR), including severe non‐proliferative diabetic retinopathy (NPDR), proliferative diabetic retinopathy (PDR) and DMO; and (4) SD‐OCT images with poor quality, rendering inaccurate assessment.

The medical records were retrieved and reviewed for baseline demographics and presence of cardiovascular comorbidities. Ophthalmic examination results, including Snellen VA and type of RVO, were recorded. Eyes with the presence of neovascularization and vitreous haemorrhage on clinical examination or non‐perfusion area on FA would be defined as ischaemic RVO. The types of RVO were classified into BRVO and CRVO; HRVO was included in the BRVO group (Ang et al., 2020; Tadayoni et al., 2016). VA at baseline, 12 and 24 months was analysed. The number and type of anti‐VEGF injections within the study period were recorded.

### 2.2 OCT images analysis

SD‐OCT (Spectralis, Heidelberg Engineering, Heidelberg, Germany) examination was performed on each patient at baseline and after completion of anti‐VEGF therapy within the 24‐month follow‐up period. All OCT images, displayed with 1:1 pixel scale, were analysed by three retinal specialists (KT, VWKH and SKH) who were masked to clinical information. The definition and grading methods of the list of quantitative and qualitative parameters were reported in Table S1.

Quantitative measurement, such as CST, was obtained by the built‐in fovea‐centred volume scan in Spectralis. The volume scans were checked for segmentation error; if present, manual correction would be performed.

Qualitative analysis of the OCT images was graded based on the horizontal scan passing through the foveal centre and the 3 B‐Scans immediately above and below the fovea. The central 1500 μm zone was analysed for the following qualitative parameters:

1. Morphology of MO
2. Vitreomacular relationship
3. Size of IRC
4. The presence of HRF
5. The presence of DRIL
6. The visibility and continuity of EZ and ELM
7. The visibility and continuity of cone outer segment tip (COST)
8. The presence of SRF

**FIGURE 1:**
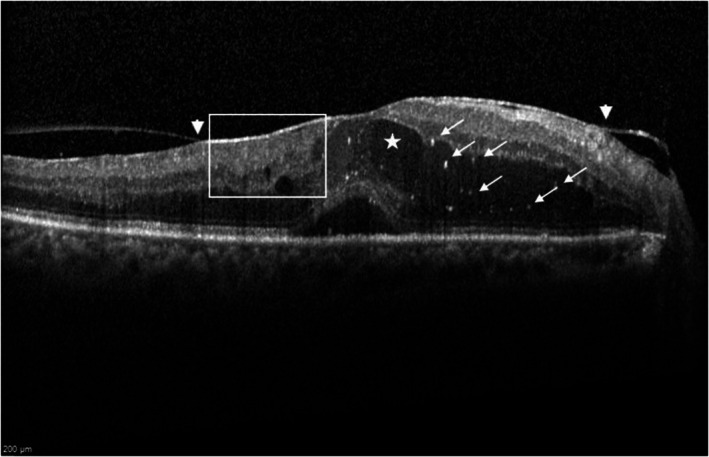
CMO + SRD morphology. Grading: ERM (white arrow heads), large IRC (white star), presence of DRIL (white box), HRF (white arrows), intact EZ/ELM.

**FIGURE 2:**
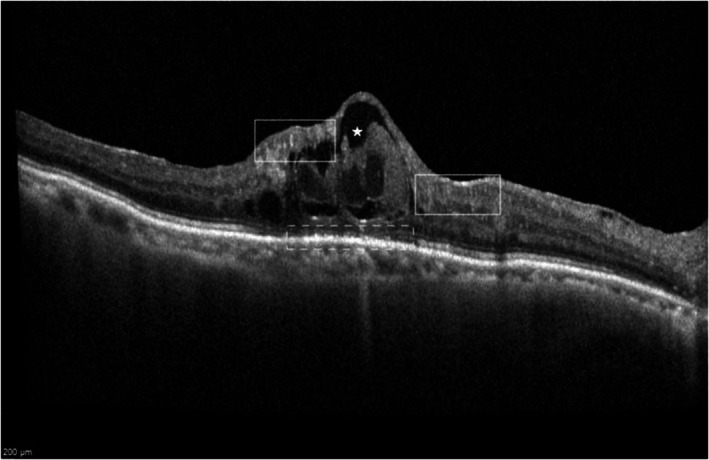
CMO morphology. Grading: No visible vitreo‐macular adhesion, large IRC (white star), presence of DRIL (white boxes), no HRF, completely disrupted EZ/ELM and COST (white dotted box).

**FIGURE 3:**
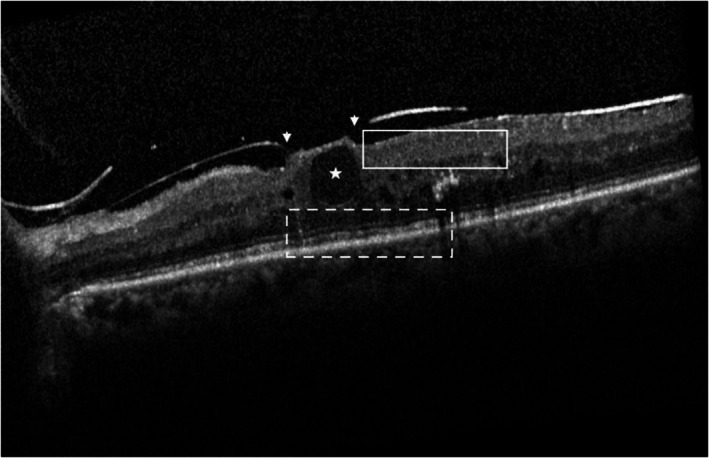
CMO morphology. Grading: VMT (white arrow heads), moderate IRC (white star), presence of DRIL (white box), no HRF, partially disrupted EZ/ELM and COST (white dotted box).

**FIGURE 4:**
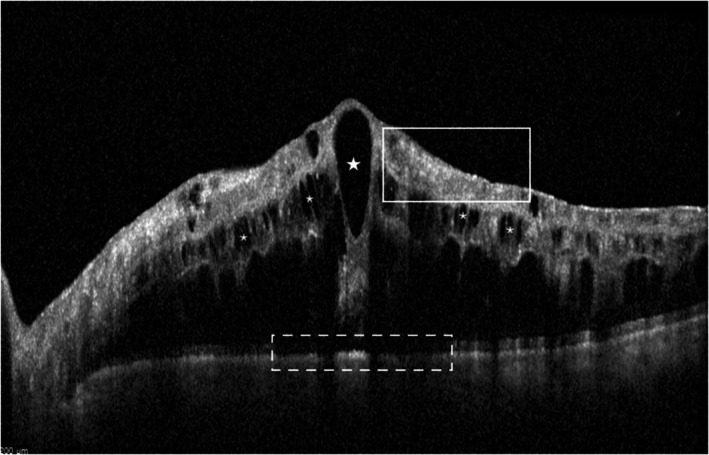
CMO + DRT morphology. Grading: No visible vitreomacular adhesion, large IRC (white stars), small IRC (white asterisks), presence of DRIL (white box), no HRF, completely disrupted EZ/ELM and COST (white dotted box).

These OCT features were graded in a semi‐quantitative manner. The morphology of MO was categorized into cystoid macular oedema (CMO), diffuse retinal thickening (DRT) and serous retinal detachment (SRD). The vitreomacular relationship was classified according to the International Vitreomacular Traction Study Group (Duker et al., 2013). The size of IRC was graded using reference images (Panozzo et al., 2020). HRF was graded into three groups: absent, 1–10 and >10 hyper‐reflective foci (Szeto, Hui, Siu, et al., 2023; Szeto, Hui, Tang, et al., 2023). DRIL was defined as the inability to differentiate the boundaries between the OPL, INL and GCL‐IPL complex and was classified as absent or present (Szeto, Hui, Siu, et al., 2023, Szeto, Hui, Tang, et al., 2023). The visibility and continuity of ELM, EZ and COST were graded as intact, disrupted and absent. We grouped EZ and ELM together for grading, as a previous study has shown that intact ELM is a pre‐requisite for intact EZ and that ELM would restore first before EZ restoration (Etheridge et al., 2021; Saxena et al., 2022). Examples for the grading of these OCT parameters are shown in Figures 1, 2, 3, 4.

**FIGURE 5:**
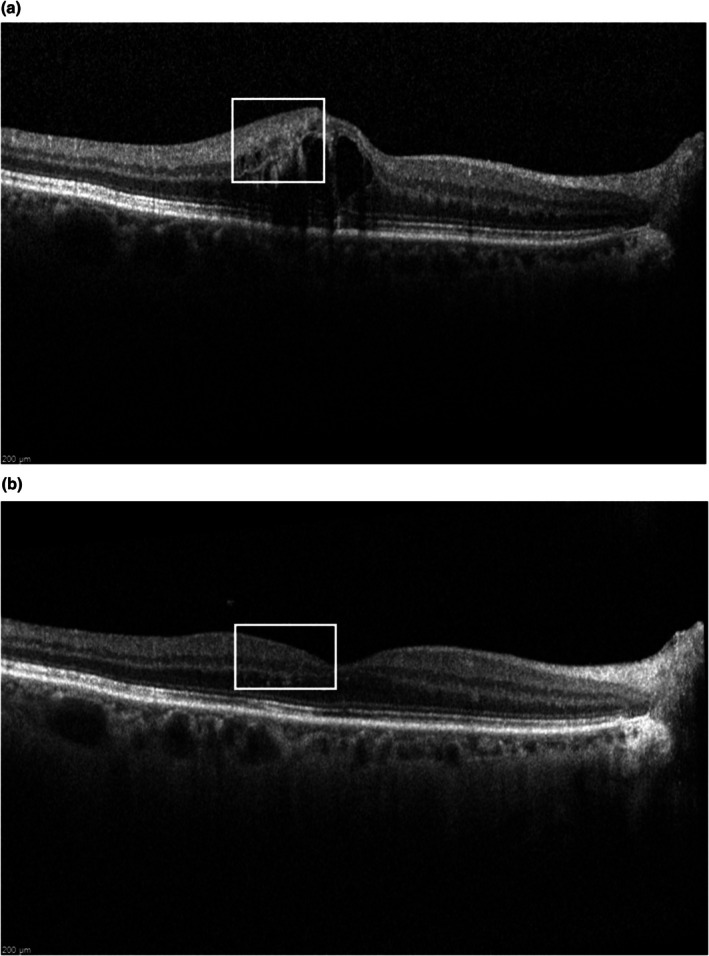
(a, b) Example of grading of DRIL in the presence of IRC over the treatment period. Presence of DRIL (white box) improves to absence of DRIL (white box). CMO, cystoid macular oedema; DRT, diffuse retinal thickening; DRIL, disorganization of retinal inner layers; ERM, epi‐retinal membrane; ELM, external limiting membrane; EZ, ellipsoid zone; IRC, intra‐retinal cyst; SRD, serous retinal detachment; VMT, vitreo‐macular traction.

Improvement in SD‐OCT biomarkers was defined as a reduction of one step or more in the severity of respective biomarkers. For example, if the ELM/EZ status of an eye changed from absent to partially disrupted, it would be considered an improvement in ELM/EZ status with treatment. Examples of improvement in DRIL before and after injection are illustrated in Figure 5a,b. Persistent MO was defined as a CST ≥320 μm after anti‐VEGF therapy (Gurudas et al., 2022).

### 2.3 Outcomes

Primary outcomes included:

- Association between baseline SD‐OCT‐based features and VA at baseline, 12 and 24 months after initiation of anti‐VEGF therapy
- Association between change in SD‐OCT‐based features with anti‐VEGF therapy and the likelihood to achieve functional success (defined as at least 5 letters gain in VA) at 12–24 months after initiation of anti‐VEGF therapy

Secondary outcome included:

- Association between baseline SD‐OCT‐based features and the likelihood to achieve at least 20% reduction in CST

### 2.4 Statistical analysis

Demographic and clinical characteristics were expressed as mean ± standard deviation for continuous variables and number of observations with percentage for categorical variables. The Shapiro‐Wilk test was used to test for normality. The independent sample *T*‐test or the Mann–Whitney *U* test was employed accordingly. The Chi‐square test was used for categorical data. Generalized estimating equation (GEE), accounting for repeated longitudinal measurement and correlated data, was performed to analyse the associations between baseline and change in SD‐OCT biomarkers with visual outcomes after anti‐VEGF treatment. Snellen VA was converted to logarithm of the Minimum Angle of Resolution (logMAR) for reporting and statistical analysis. Visual outcomes included baseline VA and change in VA at month 12 and 24 months after initiation of anti‐VEGF treatment. Univariate analysis was performed and SD‐OCT‐based biomarker with *p*‐value <0.1 would be included in multivariate analysis, with known factors that could affect post‐treatment VA, such as age, prior laser PRP treatment and presence of retinal ischaemia being included as covariates. Baseline VA was included as a covariate in the analysis change in VA (Madanagopalan & Kumari, 2018; Sophie et al., 2019; Yiu et al., 2020). Subgroup analysis was performed for treatment naive eyes.

GEE was performed to analyse the association between baseline and change in SD‐OCT measures with anatomical and functional success. Functional success was defined as a gain of 5 ETDRS letters equivalent or more in VA.

Quantitative parameters were analysed as standardized values and as continuous variables while qualitative measures were analysed as ordinal variables. Changes in qualitative parameters were categorized as binary outcomes; for example, if the EZ/ELM status changed from completely absent to partially disrupted after treatment, it would be classified as presence of improvement in EZ/ELM integrity.

The inter‐rater agreement on qualitative SD‐OCT parameters was tested with Cronbach's Alpha analysis.

All statistical analyses were performed using spss software (version 26; IBM Corp, Armonk, NY), and a *p*‐value of <0.05 is considered statistically significant in all analyses.

## 3 RESULTS

### 3.1 Baseline and follow‐up clinical characteristics and SD‐OCT‐based features

Table 1 summarized the baseline demographics, mean VA at baseline and subsequent visits, and the prevalence of each SD‐OCT‐based biomarker. One‐hundred thirty‐two eyes with RVO‐MO received anti‐VEGF treatment during the study period; 2 eyes were excluded due to prior vitreo‐retinal surgery. One‐hundred and thirty eyes were included in the final analysis. FA was performed in 24 (18.2%) eyes. Twenty‐four (18.2%) eyes were recurrent cases, of which 14 and 10 were in the BRVO and CRVO subgroup, respectively. Among recurrent cases, the mean time from last anti‐VEGF injection was 17.2 months. The mean number of injections at year 1 and year 2 was 3.85 ± 2.72 and 1.75 ± 2.32, respectively.

**TABLE 1:** Study participants' demographics, clinical characteristics and SD‐OCT based biomarkers.

	Overall (*n* = 130)	BRVO (*n* = 81)	CRVO (*n* = 49)	*p*‐Value
Age (years), means ± SD	72.22 ± 12.69	72.95 ± 11.88	71.00 ± 13.96	0.398
Gender male, *n* (%)	63 (48.5%)	42 (51.9%)	21 (42.9%)	0.320
Hypertension, *n* (%)	97 (74.6%)	58 (71.6%)	39 (79.6%)	0.311
Diabetes mellitus, *n* (%)	30 (23.1%)	15 (18.1%)	15 (30.6%)	0.113
Hyperlipidemia, *n* (%)	80 (61.5%)	47 (58.0%)	33 (67.3%)	0.290
Ischaemia, *n* (%)	28 (21.5%)	9 (11.1%)	19 (38.8%)	<0.001*
PRP, *n* (%)	26 (20.0%)	9 (11.1%)	17 (34.7%)	0.001*
Recurrent MO, *n*(%)	24 (18.5%)	14 (17.3%)	10 (20.4%)	0.816
Number of Anti‐VEGF injection, mean ± SD	5.60 ± 4.53	5.19 ± 4.48	6.29 ± 4.57	0.225
1st year	3.85 ± 2.72	3.63 ± 2.64	4.20 ± 2.84	
2nd year	1.75 ± 2.32	1.56 ± 2.33	2.08 ± 2.29	
Mean logMAR VA, mean ± SD				
Baseline	0.661 ± 0.454	0.525 ± 0.371	0.885 ± 0.492	<0.001*
6 months	0.580 ± 0.413	0.433 ± 0.295	0.824 ± 0.465	<0.001*
12 months	0.573 ± 0.432	0.419 ± 0.319	0.826 ± 0.477	<0.001*
18 months	0.567 ± 0.436	0.434 ± 0.326	0.787 ± 0.504	<0.001*
24 months	0.572 ± 0.421	0.448 ± 0.329	0.776 ± 0.477	<0.001*
Baseline OCT				
CST mean ± SD	463.52 ± 206.58	397.90 ± 118.22	572.00 ± 268.54	<0.001*
Vitreomacular relationship, *n* (%)				
No visible adhesion	85 (65.4%)	53 (65.4%)	32 (65.3%)	0.957
Incomplete vitreous detachment	14 (10.8%)	10 (12.3%)	4 (8.2%)	
Posterior vitreous detachment	2 (1.5%)	1 (1.2%)	1 (2.0%)	
Vitreomacular traction	5 (3.8%)	3 (3.7%)	2 (4.1%)	
Epiretinal membrane	24 (18.5%)	14 (17.3%)	10 (20.4%)	
IRC size, *n* (%)				
Absent	15 (11.5%)	6 (7.4%)	9 (18.4%)	<0.001*
Mild	31 (23.8%)	24 (29.6%)	7 (14.3%)	
Moderate	40 (30.8%)	32 (39.5%)	8 (16.3%)	
Severe	44 (33.8%)	19 (23.5%)	25 (51.0%)	
HRF, *n* (%)				
Absent	33 (25.4%)	19 (23.5%)	14 (28.6%)	0.516
1–10	64 (49.2%)	43 (53.1%)	21 (42.9%)	
>10	33 (25.4%)	19 (23.5%)	14 (28.6%)	
DRIL, *n* (%)				
Absent	68 (52.3%)	47 (58.0%)	21 (42.9%)	0.106
Present	62 (47.7%)	33 (42.0%)	28 (57.1%)	
EZ/ELM integrity, *n* (%)				
Intact	36 (27.7%)	26 (32.1%)	10 (20.4%)	0.001*
Disrupted	64 (49.2%)	45 (55.6%)	19 (38.8%)	
Absent	30 (23.1%)	10 (12.3%)	20 (40.8%)	
COST integrity, *n* (%)				
Intact	26 (20.0%)	19 (23.5%)	7 (14.3%)	<0.001*
Disrupted	64 (49.2%)	48 (59.3%)	16 (32.7%)	
Absent	40 (30.8%)	14 (17.3%)	26 (53.1%)	
Morphology				
DRT	42 (32.3%)	22 (27.2%)	20 (40.8%)	0.124
CMO	110 (84.6%)	70 (86.4%)	40 (81.6%)	0.617
SRD	24 (18.5%)	12 (14.8%)	12 (24.5%)	0.243

Abbreviations: BRVO, branch retinal vein occlusion; CMO, cystoid macular oedema; COST, cone outer segment tip; CRVO, central retinal vein occlusion; CST, central subfield thickness; DRIL, disorganization of retinal inner layers; DRT, diffuse retinal thickening; ELM, external limiting membrane; EZ, ellipsoid zone; HRF, hyper‐reflective foci; IRC, intra‐retinal cyst; Macular oedema, MO; PRP, pan‐retinal photocoagulation; SD‐OCT, spectral domain optical coherence tomography; SRD, serous retinal detachment; VM relationship, vitreomacular relationship.

* *p* < 0.05.

Eighty‐one (62.3%) eyes suffered from BRVO, 5 (6.0%) of which suffered from HRVO. Among the BRVO cases, 9 (11.1%) were ischaemic BRVO. Forty‐nine (37.7%) eyes suffered from CRVO, of which nineteen (38.8%) were ischaemic CRVO.

The CRVO subgroup had worse VA at baseline (0.885 vs. 0.525, *p* < 0.001), 6 (0.824 vs. 0.433, *p* < 0.001), 12 (0.826 vs. 0.419, *p* < 0.001), 18 (0.787 vs. 0.434, *p* < 0.001) and 24 (0.776 vs. 0.448, *p* < 0.001) months. There were more eyes with retinal ischaemia (38.8% vs. 11.1%, *p* < 0.001) and received laser PRP in the CRVO group (34.7% vs. 11.1%, *p* = 0.001).

The Cronbach's Alpha coefficient for inter‐rater reliability of each OCT‐based biomarker was reported in Table S2, showing acceptable to excellent internal consistency.

### 3.2 Association between baseline SD‐OCT biomarkers with baseline and subsequent VA in the BRVO subgroup

The associations between baseline OCT biomarkers and VA at baseline, 12 and 24 months for the BRVO subgroup are summarized in Table 2.

**TABLE 2:** The associations of baseline OCT measures and VA in BRVO at baseline, 12 and 24 months after initiation of anti‐VEGF injection.

VA	Baseline				12 months				24 months			
	Univariable		Multivariable		Univariable		Multivariable		Univariable		Multivariable	
Baseline biomarkers	*β* coefficient	*p* Value	*β* coefficient	*p* Value	*β* coefficient	*p* Value	*β* coefficient	*p* Value	*β* coefficient	*p* Value	*β* coefficient	*p* Value
CMO	0.166	0.088	0.052	0.610	0.052	0.633	/	/	0.048	0.638	/	/
DRT	0.028	0.751	/	/	−0.078	0.247	/	/	−0.051	0.484	/	/
SRD	0.062	0.503	/	/	−0.084	0.379	/	/	−0.059	0.557	/	/
CST^a^	0.138	0.079	0.089	0.186	0.014	0.824	/	/	0.092	0.160	/	/
IRC	0.092	0.057	0.044	0.329	0.072	0.084	0.041	0.230	0.087	0.051	0.050	0.179
HRF	0.173	0.004*	0.209	<0.001*	0.132	0.013*	0.045	0.135	0.128	0.018*	0.050	0.156
DRIL	0.140	0.082	0.182	0.012*	−0.031	0.646	/	/	−0.076	0.283	/	/
EZ/ELM	0.172	0.002*	0.144	0.008*	0.132	0.003*	0.111	0.014*	0.120	0.014*	0.096	0.042*
COST	0.208	<0.001*	0.196	<0.001*	0.096	0.032*	0.086	0.081	0.114	0.026*	0.104	0.051
VM relationship	0.006	0.804	/	/	0.023	0.279	/	/	0.026	0.241	/	/

*Note*: Multivariable analysis adjusted to age, ischemic status and PRP status.

Abbreviations: BRVO, branch retinal vein occlusion; CMO, cystoid macular oedema; COST, cone outer segment tip; CST, central subfield thickness; DRIL, disorganization of retinal inner layers; DRT, diffuse retinal thickening; ELM, external limiting membrane; EZ, ellipsoid zone; HRF, hyper‐reflective foci; IRC, intra‐retinal cyst; SRD, serous retinal detachment; VM relationship, vitreomacular relationship.

^a^ Standardized *β* coefficient reported.

* *p* < 0.05.

In multivariate analysis, the presence of HRF (*β* = 0.209, *p* < 0.001), DRIL (*β* = 0.182, *p* = 0.012), disruption of EZ/ELM (*β* = 0.144, *p* = 0.009) and disruption of COST (*β* = 0.196, *p* < 0.001) were associated with worse baseline VA. Whereas only disruption of EZ/ELM at baseline was associated with poorer VA at 12 months (*β* = 0.111, *p* = 0.014) and 24 months (*β* = 0.096, *p* = 0.042).

The morphology of MO and vitreomacular relationship was not associated with baseline or subsequent VA with treatment.

Similarly, in subgroup analysis for treatment naïve eyes, disruption of EZ/ELM at baseline was associated with poorer VA at 12 and 24 months as shown in Table S3.

### 3.3 Association between baseline SD‐OCT biomarkers with baseline and subsequent VA in the CRVO subgroup

The associations between baseline OCT biomarkers and VA at baseline, 12–24 months for the CRVO subgroup are summarized in Table 3.

**TABLE 3:** The associations of baseline OCT measures and VA in CRVO at baseline, 12 and 24 months after initiation of anti‐VEGF injection.

VA	Baseline				12 months				24 months			
	Univariable		Multivariable		Univariable		Multivariable		Univariable		Multivariable	
Baseline biomarkers	*β* coefficient	*p* Value	*β* coefficient	*p* Value	*β* coefficient	*p* Value	*β* coefficient	*p* Value	*β* coefficient	*p* Value	*β* coefficient	*p* Value
CMO	−0.056	0.680	/	/	0.017	0.931	/	/	0.290	0.012*	0.147	0.173
DRT	0.122	0.308	/	/	0.115	0.378	/	/	0.026	0.843	/	/
SRD	0.083	0.562	/	/	−0.068	0.627	/	/	−0.135	0.345	/	/
CST^a^	0.088	0.040*	0.054	0.152	0.116	0.001*	0.054	0.183	0.173	<0.001*	0.116	0.016*
IRC	0.043	0.368	/	/	0.028	0.653	/	/	0.135	0.006*	0.100	0.010*
HRF	0.043	0.638	/	/	−0.011	0.909	/	/	−0.098	0.288	/	/
DRIL	0.281	0.010*	0.231	0.032*	0.377	0.001*	0.234	0.025*	0.250	0.037*	0.152	0.162
EZ/ELM	0.343	<0.001*	0.316	<0.001*	0.379	<0.001*	0.336	<0.001*	0.372	<0.001*	0.327	<0.001*
COST	0.262	0.001*	0.234	0.001*	0.300	<0.001*	0.266	<0.001*	0.269	<0.001*	0.233	<0.001*
VM relationship	0.003	0.941	/	/	−0.007	0.855	/	/	−0.044	0.219	/	/

*Note*: Multivariable analysis adjusted to age, ischemic status and PRP.

Abbreviations: CMO, cystoid macular oedema; COST, cone outer segment tip; CRVO, central retinal vein occlusion; CST, central subfield thickness; DRIL, disorganization of retinal inner layers; DRT, Diffuse retinal thickening; ELM, external limiting membrane; EZ, ellipsoid zone; HRF, hyper‐reflective foci; IRC, intra‐retinal cyst; SRD, serous retinal detachment; VM relationship, vitreomacular relationship.

^a^ Standardized *β* coefficient reported.

* *p* < 0.05.

In multivariate analysis, the presence of DRIL (*β* = 0.231, *p* = 0.032), disruption of EZ/ELM (*β* = 0.316, *p* < 0.001) and COST (*β* = 0.234, *p* = 0.001) were associated with worse baseline VA; the presence of DRIL was associated with worse VA at 12 months (*β* = 0.234, *p* = 0.025); disruption of EZ/ELM and COST were associated with worse VA at 12 [(*β* = 0.336, *p* < 0.001) and (*β* = 0.266, *p* < 0.001)] and 24 months [(*β* = 0.327, *p* < 0.001) and (*β* = 0.233, *p* < 0.001)]; higher baseline CST (*β* = 0.115, *p* = 0.016) and larger IRC size (*β* = 0.100, *p* = 0.010) were associated with worse VA at 24 months.

The morphology of MO and vitreomacular relationship was not associated with baseline or subsequent VA with treatment.

Similarly, in treatment naïve eyes, disruption of EZ/ELM and COST at baseline was associated with poorer VA at 12 and 24 months as shown in Table S4.

### 3.4 Association between improvement in SD‐OCT parameters and subsequent VA change at 12 and 24 months

The associations between improvement in SD‐OCT measures and VA change in the BRVO and CRVO subgroups are summarized in Tables 4 and 5, respectively. The number of eyes with improvement in each biomarker and persistent MO is reported in Table S5.

**TABLE 4:** The association of improvement in OCT measures and VA change in BRVO subgroup.

OCT parameters	*n* (%)	BRVO							
		VA change at 12 months		VA gain of ≥5 letters at 12 months		VA change at 24 months		VA gain of ≥5 letters at 24 months	
		*β*	*p* Value	OR (95% CI)	*p* Value	*β*	*p* Value	OR (95% CI)	*p* Value
IRC	56/75 (74.7%)	−0.044	0.464	1.627 (0.782 to 3.383)	0.193	−0.089	0.182	1.694 (0.833 to 3.446)	0.146
HRF	22/62 (35.5%)	0.043	0.456	0.471 (0.215 to 1.032)	0.060	0.062	0.353	0.625 (0.294 to 1.326)	0.220
DRIL	10/34 (29.4%)	−0.113	0.181	4.355 (1.109 to 17.094)	0.035*	−0.137	0.097	4.510 (1.707 to 11.917)	0.002*
EZ/ELM	28/55 (50.9%)	−0.194	<0.001*	4.474 (1.783 to 11.223)	0.001*	−0.242	<0.001*	3.214 (1.414 to 7.305)	0.005*
COST	33/62 (53.2%)	−0.151	0.015*	2.778 (1.280 to 6.031)	0.010*	−0.154	0.017*	2.143 (0.998 to 4.599)	0.051

*Note*: Adjusted to baseline VA, age, ischemic status and PRP status.

Abbreviations: BRVO, branch retinal vein occlusion; CI, confidence interval; COST, cone outer segment tip; DRIL, disorganization of retinal inner layers; ELM, external limiting membrane; EZ, ellipsoid zone; HRF, hyper‐reflective foci; IRC, intra‐retinal cyst.

* *p* < 0.05.

**TABLE 5:** Association of improvement in OCT measures and VA change in the CRVO subgroup.

OCT parameters	*n* (%)	CRVO							
		VA change at 12 months		VA gain of ≥5 letters at 12 months		VA change at 24 months		VA gain of ≥5 letters at 24 months	
		*β*	*p* Value	OR (95% CI)	*p* Value	*β*	*p* Value	OR (95% CI)	*p* Value
IRC	26/40 (65.0%)	−0.160	0.102	2.959 (0.810 to 10.812)	0.101	−0.219	0.073	5.529 (1.088 to 28.094)	0.039*
HRF	9/35 (25.7%)	−0.010	0.928	0.622 (0.102 to 3.798)	0.607	−0.086	0.350	1.208 (0.371 to 3.930)	0.754
DRIL	6/28 (21.4%)	−0.205	0.209	9.507 (0.328 to 275.605)	0.190	−0.139	0.305	1.177 (0.157 to 8.802)	0.874
EZ/ELM	13/39 (33.3%)	−0.031	0.789	1.023 (0.324 to 3.228)	0.968	−0.210	0.063	1.129 (0.389 to 3.277)	0.824
COST	13/42 (31.0%)	−0.066	0.492	1.981 (0.622 to 6.311)	0.247	−0.195	0.029*	2.153 (0.785 to 5.907)	0.136

*Note*: Adjusted to baseline VA, age, ischemic status and PRP status.

Abbreviations: CI, confidence interval; COST, cone outer segment tip; CRVO, central retinal vein occlusion; DRIL, disorganization of retinal inner layers; ELM, external limiting membrane; EZ, ellipsoid zone; HRF, hyper‐reflective foci; IRC, intra‐retinal cyst.

* *p* < 0.05.

In the BRVO subgroup, there were 30 eyes (37.0%) with persistent MO. Improvement in EZ/ELM visibility was associated with a higher likelihood of achieving functional success (at least 5 letters gain in VA) at 12 (OR = 4.474, *p* = 0.001) and 24 months (OR = 3.214, *p* = 0.005) while improvement in COST visibility correlated with a higher chance of achieving functional success at 12 months (OR = 2.778, *p* = 0.010) only. Improvement in DRIL was associated with more than a 4‐fold increase in the likelihood of achieving functional success at 12 (OR = 4.355, *p* = 0.035) and 24 months (OR = 4.510, *p* = 0.002).

In the CRVO subgroup, 23 eyes (46.9%) had persistent MO. Improvement in COST was associated with greater improvement in VA at 24 months. Reduction in IRC size (OR = 5.529, 95% CI: 1.088–28.094) was significantly associated with functional success at 24 months.

### 3.5 Baseline SD‐OCT parameters as predictors of CST ≥20% reduction

The associations between baseline OCT biomarkers and the likelihood of achieving ≥20% reduction in CST after treatment are summarized in Table S6.

Larger IRC size at baseline was associated with a higher chance of achieving at least a 20% reduction in CST (OR = 4.667, *p* = 0.003) in the BRVO subgroup. No OCT‐based biomarkers were associated with the likelihood of achieving a 20% reduction in CST in the CRVO subgroup.

### 3.6 Correlation between baseline SD‐OCT parameters

The correlation between baseline SD‐OCT biomarkers is shown in Table S7. There was a weak correlation between IRC and DRIL (*r* = 0.200, *p* = 0.024), disruption of EZ/ELM (*r* = 0.238, *p* = 0.007) and COST (*r* = 0.236, *p* = 0.008). HRF correlated weakly with DRIL (*r* = 0.216, *p* = 0.019), disruption of EZ/ELM (*r* = 0.213, *p* = 0.019) and COST (*r* = 0.245, *p* = 0.007). DRIL correlated moderately with EZ/ELM disruption (*r* = 0.452, *p* < 0.001) and COST (*r* = 0.488, *p* < 0.001). EZ/ELM disruption correlated moderately to COST disruption (*r* = 0.763, *p* < 0.001).

## 4 DISCUSSION

We proposed and evaluated a clinically feasible and comprehensive SD‐OCT‐based biomarkers system in predicting the long‐term treatment response of RVO‐MO to anti‐VEGF therapy, which does not require the use of specialized software for image processing. Our findings could enhance prognosis determination, patients counselling and personalized medicine in the clinical settings.

The visibility of the EZ, ELM and COST lines on OCT corresponds to photoreceptor integrity, while DRIL represents disruption of the neural transmission pathway (Szeto et al., 2024). It is therefore sensible to observe worse baseline and subsequent VA associated with DRIL and disruption to the outer retinal layers. Reversal of the disruption to these retinal layers is possible with anti‐VEGF therapy and can be considered to be a good prognostic sign generally. The restoration of COST and EZ/ELM visibility was associated with a higher likelihood of achieving at least 5 letters gain up to 12 and 24 months in the BRVO subgroup. Resolution of DRIL with treatment was associated with increased likelihood of achieving functional success by almost 4.4‐fold at month 12 and 4.5‐fold at month 24 in the BRVO subgroup. Our findings are consistent with previous studies that change in DRIL extent during anti‐VEGF treatment correlates with vision in MO secondary to RVO (Babiuch et al., 2019; Mimouni et al., 2017).

The rates of eyes showing improved visibility of EZ/ELM and COST, and reduction in DRIL were higher in BRVO than CRVO. In addition, the proportion of eyes with reduction in DRIL (21.4%–29.4%) with anti‐VEGF therapy was also lower than our previous DMO study, of which 45.1% of patients showed improvement with anti‐VEGF therapy (Szeto, Hui, Siu, et al., 2023; Szeto, Hui, Tang, et al., 2023). The inner retina layers received metabolic supply mainly from the retinal vascular system, while the outer retina layers mainly from the choriocapillaris (CC) (Szeto et al., 2024). We hypothesize that in RVO, the retinal circulation is primarily compromised, while the choroidal circulation is relatively preserved. Therefore, DRIL is less reversible with treatment than outer retinal layers disruption. In fact, areas with DRIL were associated with flow deficit in the superficial (SCP), middle (MCP) and deep capillary plexus (DCP) on OCT‐angiography study and ischaemic index on ultra‐widefield FA (Berry et al., 2018; Onishi et al., 2019). Although secondary increase in choroidal thickness has been reported in RVO with MO, the choroidal thickening is attributed to choroidal stromal oedema while the choroidal luminal region remains relatively normal. Moreover, increased choroidal thickness may be a good prognostic factor in RVO associated MO as the choroidal circulation could maintain nutrient supply to the outer retina (Mitamura et al., 2021).

DRIL and disruption of ELM/ELM were less reversible and are less correlated with visual outcomes in the CRVO subgroup. The weaker association between OCT biomarkers and visual outcomes in CRVO could be explained by several factors. First, retinal ischaemia is more extensive and frequent in CRVO as the vascular occlusion occurs at the proximal central retinal vein near the lamina cribrosa, affecting the entire retina. While in BRVO, it occurs more distally at the arterio‐venous crossing where the retinal vein is compressed by the atherosclerotic artery (Hayreh, 2014; Scott et al., 2020). Second, there could be a “flooring effect” from poorer baseline vision in the CRVO subgroup. Third, vision is generally worse in CRVO eyes despite the resolution of MO (Hayreh, 2014). Hence, the association between change in biomarkers and VA improvement is weaker in the CRVO subgroup.

The presence of HRF at baseline was associated with worse baseline VA in the BRVO group, but not with subsequent VA. This is coherent with previous reports that HRF is not a consistent biomarker in RVO (Castro‐Navarro et al., 2022; Yiu et al., 2020). Although the nature of HRF is controversial, recent histological and proteomic studies suggested they likely represent activated microglia cells and are surrogate markers of retinal inflammation (Szeto et al., 2024; Vujosevic et al., 2024). Our findings may suggest that inflammation plays a less important role in RVO (a more acute vascular event), compared with DMO, which has a more chronic and multifactorial pathogenesis.

Although the inter‐grader agreement was acceptable to excellent in our study, an international consensus is needed for repeatable quantification of SD‐OCT‐based biomarkers (Danieli et al., 2025). For instance, the grading of DRIL requires meticulous attention in the presence of retinal oedema and IRC. Moreover, the visibility of COST is prone to the back‐shadowing effect of hard exudates and fragmentation artefact, which could be present in up to 5% of healthy eyes (Rii et al., 2012). Therefore, improvement in some biomarkers might result from the effect of the restoration of individual retinal layers or indirectly via resolution of oedema. Nevertheless, improved visibility of retinal layers, either through direct restoration of retinal strcuture or indirectly through resolution of oedema, after treatment is a good prognostic sign. Moving forward, automatic artificial intelligence (AI)‐assisted grading of SD‐OCT biomarkers may be a promising tool to reliably and reproducibly quantify these biomarkers (Danieli et al., 2025).

We investigated a comprehensive SD‐OCT‐based biomarkers system that is practical to use in a clinical setting to predict the long‐term treatment response of RVO‐MO. Previously, there were inconsistent conclusions on OCT biomarkers in predicting the functional outcome of RVO‐MO, which may be explained by heterogeneous study designs, inclusion of eyes treated with both anti‐VEGF and steroid, unreported ischaemic status, and lack of longitudinal analysis of SD‐OCT‐based biomarkers (Castro‐Navarro et al., 2022; Yiu et al., 2020). Our findings highlight the importance of evaluating the longitudinal change in SD‐OCT‐based biomarkers as improvement in DRIL, EZ/ELM and COST status after anti‐VEGF therapy increased the odds of significant VA improvement by 24 months. The inclusion of long‐term follow‐up data up to 24 months is clinically important. In contrast, most publications had follow‐up periods of 7–12 months (Babiuch et al., 2019; Castro‐Navarro et al., 2022; Mimouni et al., 2017; Yiu et al., 2020). In fact, the mean number of anti‐VEGF injections between month 6 and month 24 could vary from 11.3 in RCT to 2.5 in real‐world practice (Ang et al., 2020; Tadayoni et al., 2017). Additionally, the grading system we evaluated avoids the time‐consuming process of manual measurement of each individual biomarker; hence, it could be practical and intuitive for clinicians to apply in retina clinics. Last, correlation between different SD‐OCT features was present in our cohort and prior studies (Szeto et al., 2024; Szeto, Hui, Siu, et al., 2023; Szeto, Hui, Tang, et al., 2023). Therefore, previous publications that concluded no association between SD‐OCT features and visual outcomes may be confounded by multi‐collinearity (Yiu et al., 2020). This potential confounder was minimized in our study as each SD‐OCT feature was analysed individually, controlling for known factors affecting VA (such as age, ischaemic status, baseline VA and laser PRP treatment).

Limitations of this study include its retrospective nature. Second, both treatment naïve and previously treated patients were included. Third, the mean number of injections was relatively low, partially because the study was conducted during the COVID period. Regardless, in a real‐world clinical setting, a systematic review showed that the mean number of injections was 5.2 in 24 months, which is consistent with our cohort and considerably lower than prospective RCTs (Ang et al., 2020). Future studies with pre‐defined treatment regimens are required to extrapolate our findings to high resource settings with adherence to intensive treatment regimens. It is worth noting that the rate of persistent MO in our cohort is comparable to that in RCTs. In fact, 10.7% and 60.8% of eyes had persistent and recurrent MO, respectively, in the LEAVO study, and Ciulla et al. reported that 28.3% of eyes had persistent or worsening of IRC in the post‐hoc analysis of 3 RCTs (Ciulla et al., 2022; Gurudas et al., 2022). Thus, we believe these SD‐OCT‐based biomarkers would still possess prognostic implications under more aggressive treatment regimens. Fourth, HRVO was included in the BRVO group. We acknowledge that there is currently no consensus on the classification of HRVO (Ang et al., 2020); our classification was justified by a recent study which showed that eyes with HRVO showed similar VA as BRVO after 12 months of anti‐VEGF therapy (Hunt et al., 2023). Last, FA was performed in selected cases due to the limited resources. As a result, the non‐perfusion area was not measured, and there could be an underestimation of the prevalence of ischaemia in our cohort. In the context of RVO‐MO, the presence of macular capillary non‐perfusion on FA was not correlated with post‐treatment BCVA and injection frequency in the analysis of several RCTs (Ciulla et al., 2022; Tadayoni et al., 2017). Despite these limitations, we believe our study is relevant in the real‐world clinical setting, where ophthalmologists need to treat both naïve and recurrent cases with limited resources and access to invasive investigations such as FA (Ang et al., 2020; Laouri et al., 2011). These limitations could be addressed in future studies with standardized grading and quantification of biomarkers and pre‐defined treatment intensity. Moreover, the inclusion of enhanced depth imaging (EDI) and OCTA in future studies may provide more insight into the pathogenesis and prognosis of RVO‐MO.

## 5 CONCLUSION

SD‐OCT‐based biomarkers could inform the prognosis and functional response of MO secondary to RVO treated with anti‐VEGF therapy. Disrupted EZ/ ELM at baseline is a robust biomarker for less VA gain with anti‐VEGF therapy in both BRVO and CRVO subgroups through 2 years. The presence of DRIL at baseline predicted less VA gain with therapy in the CRVO subgroup. Nevertheless, both disrupted EZ/ELM and DRIL could be reversible with anti‐VEGF therapy, and improvement of these biomarkers correlated with greater VA gain in the BRVO subgroup but not in the CRVO subgroup, highlighting the importance of distinguishing BRVO from CRVO in clinical practice and future research. Our findings support the use of SD‐OCT‐based biomarkers to improve patients counselling on prognosis of MO secondary to RVO with anti‐VEGF treatment in the clinical setting.

## FUNDING INFORMATION

This study received no funding.

## CONFLICT OF INTEREST STATEMENT

The authors declare no competing interests in relation to the study.

## DISCLOSURES

Simon K.H. Szeto received consultant fees from Roche and Astella. Timothy Y.Y. Lai received funding from Allergan, Bayer, Novartis and Roche, as well as consultant/ personal fees from Allergan, Bayer, Boehringer Ingelheim, Chengdu Kanghong Biotechnology, Iveric Bio, Novartis, Oculis and Roche.

## Supporting information


Table S1.

Table S2.

Table S3.

Table S4.

Table S5.

Table S6.

Table S7.
