# Supplementary material for: SD‐OCT‐based biomarkers in predicting treatment outcomes of macular oedema secondary to retinal vein occlusion treated with anti‐VEGF therapy
Source: Acta Ophthalmol. 2025 Aug 4;104(2):e152–64. doi: 10.1111/aos.17574 (PMC12888950; doi:10.1111/aos.17574)
Supplement: Supplementary file 1 — Table S1. [file AOS-104-e152-s003.docx]

**Supplementary Table 1:** Definition and Grading of SD-OCT based parameters

|  | Definition | Grading |
| --- | --- | --- |
| CST | Provided by Spectralis built-in software by volume scan | Measured in µm |
| Morphology of MO |  |  |
| CMO | Presence of intraretinal cystoid spaces | Absent; Present |
| DRT | Diffuse sponge like thickening of the retina | Absent; Present |
| SRD | Presence of subretinal fluid | Absent; Present |
| Vitreomacular relationship | Anatomical classification looking at degree of separation or traction between vitreous and macula on OCT image. | No visible adhesion; Incomplete vitreous detachment; Complete posterior vitreous detachment; Vitreomacular traction; Epiretinal membrane |
| IRC | Hyporeflective cavities within External liming membrane and Internal limiting membrane | Absent; Mild; Moderate, Severe |
| HRF | Hyper-reflective foci, with similar reflectivity to RPE layer, located between Internal Limiting Membrane and RPE layer. Characterized by absence of back shadowing and <30 µm in diameter. Grading based on the 1500µm zone of the horizontal scan centred on the fovea | Absent; 1-10; >10 |
| DRIL | Indiscernible boundaries between Ganglion Cell- Inner Plexiform Layer complex, Inner Nuclear Later and Outer Plexiform Layer. Graded based on the central 1mm zone of horizontal scan centred on fovea. | Absent; Present |
| EZ/ELM | The first and second hyper-reflective bands of the outermost four layers on OCT. Graded within the central 1mm wide area of the horizontal B-scan centred on fovea centre | Intact = whole band is visible; Disrupted = visible band but with partial interruption; Absent = No discernable band |
| COST | The third hyper-reflective band of the outermost four layers on OCT. Graded within the central 1mm wide area of the horizontal B-scan centred on fovea centre | Intact = whole band is visible; Disrupted = visible band but with partial interruption; Absent = No discernable band |
| SRF | Presence of fluid underneath neurosensory retina | Absent; Present |

CMO: cystoid macular oedema; COST: cone outer segment tip; CST: central subfield thickness; DRIL: disorganization of retinal inner layers; DRT: diffuse retinal thickening; ELM: external limiting membrane; EZ: ellipsoid zone; HRF: hyper-reflective foci; IRC: intra-retinal cyst; SD-OCT: spectral domain optical coherence tomography; SRD: serous retinal detachment
