# Supplementary material for: SD‐OCT‐based biomarkers in predicting treatment outcomes of macular oedema secondary to retinal vein occlusion treated with anti‐VEGF therapy
Source: Acta Ophthalmol. 2025 Aug 4;104(2):e152–64. doi: 10.1111/aos.17574 (PMC12888950; doi:10.1111/aos.17574)
Supplement: Supplementary file 2 — Table S2. [file AOS-104-e152-s005.docx]

**Supplementary Table 2:** Inter-grader agreement for SD-OCT based parameters

|  | IRC | HRF | DRIL | EZ/ELM | COST | SRF | VM relationship |
| --- | --- | --- | --- | --- | --- | --- | --- |
| Cronbach’s Alpha coefficient | 0.959 | 0.718 | 0.848 | 0.944 | 0.881 | 1.000 | 0.881 |

COST: cone outer segment tip; DRIL: disorganization of retinal inner layers; ELM: external limiting membrane; EZ: ellipsoid zone; HRF: hyper-reflective foci; IRC: Intra-retinal cyst; SD-OCT: spectral domain optical coherence tomography; SRF: sub-retinal fluid; VM relationship: vitreomacular relationship
