# Supplementary material for: SD‐OCT‐based biomarkers in predicting treatment outcomes of macular oedema secondary to retinal vein occlusion treated with anti‐VEGF therapy
Source: Acta Ophthalmol. 2025 Aug 4;104(2):e152–64. doi: 10.1111/aos.17574 (PMC12888950; doi:10.1111/aos.17574)
Supplement: Supplementary file 3 — Table S3. [file AOS-104-e152-s007.docx]

**Supplementary Table 3** The associations of baseline OCT measures and VA in BRVO at baseline, 12 and 24 months after initiation of anti-VEGF injection in eye without recurrence

| VA | Baseline | | | | 12 months | | | | 24 months | | | | |
| --- | --- | --- | --- | --- | --- | --- | --- | --- | --- | --- | --- | --- | --- |
|  | Univariable | | Multivariable | | Univariable | | Multivariable | | Univariable | | Multivariable | |  |
| Baseline biomarkers | β coefficient | P value | β coefficient | P value | β coefficient | P value | β coefficient | P value | β coefficient | P value | β coefficient | P value |  |
| CMO | 0.144 | 0.171 | / | / | 0.039 | 0.745 | / | / | 0.061 | 0.578 | / | / |  |
| DRT | 0.077 | 0.409 | / | / | -0.035 | 0.640 | / | / | -0.004 | 0.963 | / | / |  |
| SRD | 0.067 | 0.509 | / | / | -0.088 | 0.400 | / | / | -0.103 | 0.282 | / | / |  |
| CST# | 0.152 | 0.097 | 0.103 | 0.156 | 0.019 | 0.799 | / | / | 0.060 | 0.405 | / | / |  |
| IRC | 0.103 | 0.079 | 0.051 | 0.338 | 0.074 | 0.150 | / | / | 0.078 | 0.130 | / | / |  |
| HRF | 0.188 | 0.006* | 0.223 | <0.001* | 0.154 | 0.012* | 0.042 | 0.210 | 0.140 | 0.020* | 0.041 | 0.275 |  |
| DRIL | 0.090 | 0.310 | / | / | -0.065 | 0.400 | / | / | -0.088 | 0.258 | / | / |  |
| EZ/ELM | 0.167 | 0.008^*^ | 0.149 | 0.016* | 0.125 | 0.017^*^ | 0.113 | 0.036^*^ | 0.114 | 0.030^*^ | 0.101 | 0.047* |  |
| COST | 0.200 | <0.001^*^ | 0.201 | 0.001* | 0.109 | 0.037^*^ | 0.107 | 0.063 | 0.092 | 0.097 | 0.092 | 0.127 |  |
| VM relationship | 0.005 | 0.867 | / | / | 0.033 | 0.222 | / | / | 0.029 | 0.250 | / | / |  |

BRVO: branch retinal vein occlusion; CMO: cystoid macular oedema; COST: cone outer segment tip; CST: central subfield thickness; DRIL: disorganization of retinal inner layers; DRT: Diffuse retinal thickening; ELM: external limiting membrane; EZ: ellipsoid zone; HRF: hyper-reflective foci; IRC: intra-retinal cyst; SRD: serous retinal detachment; VM relationship: Vitreomacular relationship

Multivariable analysis adjusted to age, ischemic status and PRP status

*p<0.05

#standardised β coefficient reported
