# Supplementary material for: SD‐OCT‐based biomarkers in predicting treatment outcomes of macular oedema secondary to retinal vein occlusion treated with anti‐VEGF therapy
Source: Acta Ophthalmol. 2025 Aug 4;104(2):e152–64. doi: 10.1111/aos.17574 (PMC12888950; doi:10.1111/aos.17574)
Supplement: Supplementary file 4 — Table S4. [file AOS-104-e152-s004.docx]

**Supplementary Table 4** The associations of baseline OCT measures and VA in CRVO at baseline, 12 and 24 months after initiation of anti-VEGF injection in eye without recurrence

| VA | Baseline | | | | 12 months | | | | 24 months | | | | |
| --- | --- | --- | --- | --- | --- | --- | --- | --- | --- | --- | --- | --- | --- |
|  | Univariable | | Multivariable | | Univariable | | Multivariable | | Univariable | | Multivariable | | |
| Baseline biomarkers | β coefficient | P value | β coefficient | P value | β coefficient | P value | β coefficient | P value | β coefficient | P value | β coefficient | P value |  |
| CMO | -0.061 | 0.728 | / | / | -0.022 | 0.930 | / | / | 0.250 | 0.081 | 0.110 | 0.374 |  |
| DRT | 0.196 | 0.243 | / | / | 0.162 | 0.347 | / | / | 0.086 | 0.602 | / | / |  |
| SRD | 0.130 | 0.545 | / | / | -0.072 | 0.722 | / | / | -0.094 | 0.636 | / | / |  |
| CST# | 0.112 | 0.064 | 0.054 | 0.235 | 0.137 | 0.001* | 0.069 | 0.096 | 0.183 | 0.004* | 0.125 | 0.010* |  |
| IRC | 0.047 | 0.456 | / | / | 0.013 | 0.867 | / | / | 0.127 | 0.030* | 0.092 | 0.029* |  |
| HRF | 0.100 | 0.416 | / | / | 0.025 | 0.832 | / | / | -0.043 | 0.700 | / | / |  |
| DRIL | 0.411 | 0.003^*^ | 0.245 | 0.055 | 0.421 | 0.002^*^ | 0.200 | 0.068 | 0.329 | 0.021^*^ | 0.126 | 0.298 |  |
| EZ/ELM | 0.443 | <0.001^*^ | 0.331 | 0.001^*^ | 0.430 | <0.001^*^ | 0.290 | <0.001^*^ | 0.398 | <0.001^*^ | 0.309 | <0.001^*^ |  |
| COST | 0.328 | 0.001^*^ | 0.199 | 0.037^*^ | 0.352 | <0.001^*^ | 0.202 | 0.003^*^ | 0.307 | <0.001^*^ | 0.183 | 0.017^*^ |  |
| VM relationship | -0.012 | 0.817 | / | / | -0.013 | 0.829 | / | / | -0.039 | 0.398 | / | / |  |

CMO: cystoid macular oedema; COST: cone outer segment tip; CRVO: central retinal vein occlusion; CST: central subfield thickness; DRIL: disorganization of retinal inner layers; DRT: Diffuse retinal thickening; ELM: external limiting membrane; EZ: ellipsoid zone; HRF: hyper-reflective foci; IRC: intra-retinal cyst; SRD: serous retinal detachment; VM relationship: vitreomacular relationship

Multivariable analysis adjusted to age, ischemic status and PRP

*p<0.05

#standardised β coefficient reported
