# Supplementary material for: SD‐OCT‐based biomarkers in predicting treatment outcomes of macular oedema secondary to retinal vein occlusion treated with anti‐VEGF therapy
Source: Acta Ophthalmol. 2025 Aug 4;104(2):e152–64. doi: 10.1111/aos.17574 (PMC12888950; doi:10.1111/aos.17574)
Supplement: Supplementary file 5 — Table S5. [file AOS-104-e152-s002.docx]

**Supplementary Table 5:** Number of eyes with improvement in OCT parameters and persistent macular oedema

|  | BRVO n, (%) | CRVO n, (%) |
| --- | --- | --- |
| Improvement in OCT parameters |  |  |
| IRC | 56/75 (74.7 %) | 26/40 (65.0 %) |
| HRF | 22/62 (35.5 %) | 9/35 (25.7 %) |
| DRIL | 10/34 (29.4 %) | 6/28 (21.4 %) |
| EZ/ELM | 28/55 (50.9 %) | 13/39 (33.3 %) |
| COST | 33/62 (53.2 %) | 13/42 (31.0 %) |
| Persistent Macular Oedema | 30/81 (37.0 %) | 23/49 (46.9 %) |

BRVO: branch retinal vein occlusion; COST: cone outer segment tip; CRVO: central retinal vein occlusion; DRIL: disorganization of retinal inner layers; ELM: external limiting membrane; EZ: ellipsoid zone; HRF: hyper-reflective foci; IRC: intra-retinal cyst;
