# Supplementary material for: SD‐OCT‐based biomarkers in predicting treatment outcomes of macular oedema secondary to retinal vein occlusion treated with anti‐VEGF therapy
Source: Acta Ophthalmol. 2025 Aug 4;104(2):e152–64. doi: 10.1111/aos.17574 (PMC12888950; doi:10.1111/aos.17574)
Supplement: Supplementary file 6 — Table S6. [file AOS-104-e152-s006.docx]

**Supplementary Table 6:** Logistic regression of CST ≥20% reduction and baseline OCT biomarkers

|  | BRVO | |  |  | CRVO | |  |
| --- | --- | --- | --- | --- | --- | --- | --- |
| Baseline OCT biomarkers | OR (95% CI)^#^ | p value | OR (95% CI)^#^ | | | p value | |
| IRC | 4.667 (1.717 – 12.684) | 0.003* | 1.405 (0.696 – 2.839) | | | 0.343 | |
| HRF | 0.691 (0.269 – 1.774) | 0.442 | 1.064 (0.476 – 2.381) | | | 0.476 | |
| DRIL | 1.765 (0.569 – 5.469) | 0.325 | 0.924 (0.238 – 3.582) | | | 0.908 | |
| EZ/ELM | 1.235 (0.518 – 2.947) | 0.634 | 0.879 (0.386 – 2.000) | | | 0.759 | |
| COST | 1.091 (0.407 – 2.926) | 0.863 | 0.554 (0.242 – 1.269) | | | 0.163 | |

BRVO: branch retinal vein occlusion; CI: confidence interval; COST: cone outer segment tip; CRVO: central retinal vein occlusion; CST: central subfield thickness; DRIL: disorganised retinal inner layers; ELM: external limiting membrane; EZ: ellipsoid zone; HRF: hyper-reflective foci; IRC: intra-retinal cyst;

#Adjusted to Age, baseline CST, ischaemic status and prior PRP treatment

*p<0.05
