# Supplementary material for: SD‐OCT‐based biomarkers in predicting treatment outcomes of macular oedema secondary to retinal vein occlusion treated with anti‐VEGF therapy
Source: Acta Ophthalmol. 2025 Aug 4;104(2):e152–64. doi: 10.1111/aos.17574 (PMC12888950; doi:10.1111/aos.17574)
Supplement: Supplementary file 7 — Table S7. [file AOS-104-e152-s001.docx]

**Supplementary Table 7:** Correlation between baseline OCT parameters

| OCT biomarkers | IRC,  r (p-value) | HRF,  r (p-value) | DRIL,  r (p-value) | EZ/ELM,  r (p-value) | COST,  r (p-value) |
| --- | --- | --- | --- | --- | --- |
| IRC,  r (p-value) |  | 0.085 (p=0.364) | 0.200 (p=0.024^*^) | 0.238 (p=0.007^*^) | 0.236 (p=0.008^*^) |
| HRF,  r (p-value) | 0.085 (p=0.364) |  | 0.216 (p=0.019^*^) | 0.213 (p=0.019^*^) | 0.245 (P=0.007*) |
| DRIL,  r (p-value) | 0.200 (p=0.024^*^) | 0.216 (p=0.019^*^) |  | 0.452 (p<0.001^*^) | 0.488 (p<0.001^*^) |
| EZ/ELM,  r (p-value) | 0.238 (p=0.007^*^) | 0.213 (p=0.019^*^) | 0.452 (p<0.001^*^) |  | 0.763 (p<0.001^*^) |
| COST,  r (p-value) | 0.236 (p=0.008^*^) | 0.245 (P=0.007*) | 0.488 (p<0.001^*^) | 0.763 (p<0.001^*^) |  |

COST: cone outer segment tip; CST: central subfield thickness; DRIL: disorganization of retinal inner layers; ELM: external limiting membrane; EZ: ellipsoid zone; HRF: hyper-reflective foci; IRC: intra-retinal cyst;

*p<0.05

r: Pearson correlation coefficient
